# Pre-clinical quantitative imaging and mouse-specific dosimetry for ^111^In-labelled radiotracers

**DOI:** 10.1186/s13550-016-0238-z

**Published:** 2016-11-25

**Authors:** Ana M. Denis-Bacelar, Sarah E. Cronin, Chiara Da Pieve, Rowena L. Paul, Sue A. Eccles, Terence J. Spinks, Carol Box, Adrian Hall, Jane K. Sosabowski, Gabriela Kramer-Marek, Glenn D. Flux

**Affiliations:** 1 Joint Department of Physics, The Institute of Cancer Research and The Royal Marsden Hospital NHS Foundation Trust, London, SM2 5NG United Kingdom; 2 Division of Cancer Therapeutics, The Institute of Cancer Research, London, SM2 5NG United Kingdom; 3 Radiopharmacy Department, The Royal Marsden Hospital NHS Foundation Trust, London, SM2 5PT United Kingdom; 4 Centre for Molecular Oncology, Barts Cancer Institute, Queen Mary University of London, London, EC1M 6BQ United Kingdom; 5 Division of Radiotherapy and Imaging, The Institute of Cancer Research, London, SM2 5NG United Kingdom

**Keywords:** ^111^In, Pre-clinical, Image quantification, Partial volume effect, Dosimetry, Radiolabelled antibodies, SPECT, HER2

## Abstract

**Background:**

Accurate quantification in molecular imaging is essential to improve the assessment of novel drugs and compare the radiobiological effects of therapeutic agents prior to in-human studies. The aim of this study was to investigate the challenges and feasibility of pre-clinical quantitative imaging and mouse-specific dosimetry of ^111^In-labelled radiotracers.

Attenuation, scatter and partial volume effects were studied using phantom experiments, and an activity calibration curve was obtained for varying sphere sizes. Six SK-OV-3-tumour bearing mice were injected with ^111^In-labelled HER2-targeting monoclonal antibodies (mAbs) (range 5.58–8.52 MBq). Sequential SPECT imaging up to 197 h post-injection was performed using the Albira SPECT/PET/CT pre-clinical scanner. Mice were culled for quantitative analysis of biodistribution studies. The tumour activity, mass and percentage of injected activity per gram of tissue (%IA/g) were calculated at the final scan time point and compared to the values determined from the biodistribution data. Delivered ^111^In-labelled mAbs tumour absorbed doses were calculated using mouse-specific convolution dosimetry, and absorbed doses for ^90^Y-labelled mAbs were extrapolated under the assumptions of equivalent injected activities, biological half-lives and uptake distributions as for ^111^In.

**Results:**

For the sphere sizes investigated (volume 0.03–1.17 ml), the calibration factor varied by a factor of 3.7, whilst for the range of tumour masses in the mice (41–232 mg), the calibration factor changed by a factor of 2.5. Comparisons between the mice imaging and the biodistribution results showed a statistically significant correlation for the tumour activity (*r* = 0.999, *P* < 0.0001) and the tumour mass calculations (*r* = 0.977, *P* = 0.0008), whilst no correlation was found for the %IA/g (*r* = 0.521, *P* = 0.29). Median tumour-absorbed doses per injected activity of 52 cGy/MBq (range 36–69 cGy/MBq) and 649 cGy/MBq (range 441–950 cGy/MBq) were delivered by ^111^In-labelled mAbs and extrapolated for ^90^Y-labelled mAbs, respectively.

**Conclusions:**

This study demonstrates the need for multidisciplinary efforts to standardise imaging and dosimetry protocols in pre-clinical imaging. Accurate image quantification can improve the calculation of the activity, %IA/g and absorbed dose. Diagnostic imaging could be used to estimate the injected activities required for therapeutic studies, potentially reducing the number of animals used.

## Background

Molecular imaging enables minimally-invasive visualisation of molecular and cellular biological processes in living organisms. It plays an important role in cancer drug development and in monitoring disease progression and tumour response to therapeutic interventions [1–3]. Animal models are both cost effective and versatile and therefore have been essential in cancer research. Ex vivo biodistribution and/or autoradiography studies are traditionally used to investigate the uptake characteristics of novel radiolabelled tracers prior to translation to in-human clinical trials. However, these methods are limited, as they require animals to be culled at various time points and the pharmacokinetics are based on data from different animals at different times. Conversely, SPECT and PET pre-clinical imaging enables the prospect of longitudinal studies and therefore has the potential to provide quantitative measurements of radiotracer biodistribution and to reduce the number of animals required per study, which is both more cost effective and more ethical than traditional methods [4].

Absolute image quantification is essential to evaluate imaging biomarkers and to accurately determine the distribution of the uptake of novel radiotracers to evaluate their toxicity and efficacy profile in small animals prior to use in human studies. It is also necessary for dosimetry calculations and therefore has the potential to improve our understanding of the biological mechanisms of radiation-induced cell damage [5, 6] and to better inform the comparison of therapeutic radiotracers. The accuracy of pre-clinical molecular imaging can be degraded by several factors including attenuation, scatter, partial volume, motion and animal handling [7]. The effects of attenuation and scatter are of less importance than in clinical imaging due to the smaller size of the subjects involved. However, multiple studies have shown that the effects can be significant for radionuclides emitting low-energy gamma rays and larger-sized rodents [8–13]. The spatial resolution of the imaging system also affects quantification due to the partial volume effect, particularly in the case of small animals. The majority of studies to investigate partial volume effects in pre-clinical imaging have focused on PET [14–16]. Few correction methods are available [17] and commercial imaging systems do not provide correction and/or compensation methods for partial volume effects.

The aim of this study was to explore the challenges and potential role of quantitative pre-clinical SPECT imaging as an alternative for biodistribution studies. Methods used in the clinical setting were applied to a pre-clinical study to investigate image quantification and mouse-specific dosimetry of ^111^In-labelled monoclonal antibodies (mAbs) targeting HER2-positive tumours. In particular, the influence of tumour size on quantification accuracy was investigated.

## Methods

### Immunoconjugate preparation and radiolabelling

The HER2-targeting mAbs used in this study were the commercially available trastuzumab (Herceptin®, Roche) and ICR12, developed at The Institute of Cancer Research, London [18, 19]. The bifunctional chelator 2-(4-isothiocyanatobenzyl)-diethylenetriaminepentaacetic acid (pSCN-Bn-DTPA, Macrocyclics, US) was conjugated to ICR12 and trastuzumab. Additionally, 2-(4-isothiocyanatobenzyl)-1, 4, 7, 10-tetraazacyclododecane-1, 4, 7, 10-tetraacetic acid (p-SCN-Bn-DOTA, Macrocyclics, US) was conjugated to trastuzumab. The immunoconjugates (50–80 μg) were radiolabelled with ^111^In (*ca* 42 MBq) (Perking Elmer, US) in acetate buffer (pH = 4). All reactions were performed as described previously [20, 21]. The use of different radioimmunoconjugates does not affect the results of this study, as the primary aims were to investigate the feasibility and challenges of image quantification and dosimetry in pre-clinical studies, rather than to compare the radiotracers.

### Tumour cell line and animals

All experiments were performed in compliance with licences issued under the UK Animals (Scientific Procedures) Act 1986 following local ethical review and the United Kingdom National Cancer Research Institute Guidelines for Animal Welfare in Cancer Research [4]. Six female CD-1 nude mice (6–8-week-old) were injected subcutaneously on the flank with high HER2-overexpressing SK-OV-3 human ovarian carcinoma cells (5 × 10^6^/mouse) (ATCC, USA) suspended in 30% Matrigel (BD Biosciences, UK) diluted in HBSS (Gibco, Thermo Scientific, UK). Tumours were allowed to grow for 3–4 weeks and the radioimmunoconjugates, diluted in saline, were injected via the tail vein (activity range 5.58–8.52 MBq/mouse, quantity of antibody range 8–25 μg) (see Table 1).

**Table 1:** Radioimmonucongugate, level of activity (A_inj_) and quantity of antibody (mAb_inj_) injected for the six mice included in the study

Mouse no.	Radioimmonoconjugate	A_inj_ (MBq)	mAb_inj_ (μg)
M1	^111^In-DTPA-trastuzumab	6.14	12
M2	^111^In-DTPA-ICR12	6.39	12
M3	^111^In-DTPA-ICR12	5.58	10
M4	^111^In-DOTA-trastuzumab	8.52	25
M5	^111^In-DOTA-trastuzumab	8.32	8
M6	^111^In-DTPA-ICR12	7.16	10

### Imaging study

Mice were anesthetised by inhalation of a 2% isoflurane/O_2_ mixture (Virbac, UK) and placed on the scanner bed. A 1% isoflurane gas mixture was maintained during acquisition. The imaging study was performed using the Albira SPECT/PET/CT tri-modal pre-clinical scanner (Bruker), which allows fully registered anatomical and functional imaging [22, 23]. The CT component is co-planar with the SPECT crystals and has transverse and axial fields of view (FOV) of 65 mm. The SPECT sub-system comprises two opposing heads, each with a single 100 × 100 × 4 mm^2^ CsI(Na) crystal and a position sensitive photomultiplier tube. All acquisitions were performed with a single-pinhole collimator and transverse and axial FOVs of 80 mm and 60 mm, respectively. An energy window width of 20% was placed on the lower gamma-ray emission of ^111^In at 171 keV. The scans were reconstructed using default parameters, the ordered subsets expectation maximization (OSEM) method with two iterations and five subsets into a 110 × 110 matrix with voxel size of 0.75 mm. The images were not corrected for attenuation, scatter or partial volume effects, as these are not provided by the manufacturer. The six mice were imaged at 4, 24, 48, 72 and 96 h post-injection (pi), and two mice were additionally imaged at 168 and 197 h post-injection. A CT (45 kV, 0.2 mA, 80 mSv) and a 15 min SPECT acquisition were performed at each time point.

#### Image quantification

In-built attenuation and scatter corrections are not available in the Albira system. Therefore, a simple phantom experiment was used to investigate their combined effect. A 2.3 mm-radius sphere was filled with 32.6 MBq of ^111^In (0.1 HCl) and attached to a 30.4 ml cylindrical phantom (diameter 25 mm, length 92 mm) to simulate a subcutaneous tumour in the flank of a mouse. The phantom was placed on a moveable platform adjustable to sub-millimetre precision and scanned in ten positions: five axial positions in the centre and five positions 8 mm off-centre from the FOV trans-axially. The experiment was performed with the cylindrical phantom filled with air and water. The mean counts in the sphere were compared for both cases to assess the effects of attenuation and scatter within the mice.

To quantify the level of activity in the in vivo SPECT scans, phantom studies were performed to convert from measured counts to units of activity (MBq). Three spherical phantoms with volumes of 0.03, 0.12 and 1.17 ml were filled with activity concentrations in the range of 7.7–10.2 MBq/ml of ^111^In (0.1 M HCl) to investigate partial volume effects and to calibrate the SPECT images. Each sphere was attached to a 30.4 ml cylindrical phantom (diameter 25 mm, length 92 mm) filled with 0.1 M HCl solution to provide a realistic scatter and attenuation medium.

Each phantom was scanned using a 15-min acquisition and reconstructed with the same parameters as for the in vivo study.

#### Dosimetry

Image-based convolution dosimetry was performed using in-house software [24], developed in C# and C++, using the .Net framework and the open source library Visualization Toolkit VTK (http://www.vtk.org) [25]. Three-dimensional absorbed dose distributions were obtained from the convolution of a voxelised-cumulated activity distribution and a voxel *S*-value kernel. The SPECT scans were co-registered using a rigid mutual information algorithm, and these were used to obtain the time-activity curves and to calculate the cumulated activity at the voxel level. A trapezoidal or exponential extrapolation method was used for the intermediate phases, depending on whether the activity between the two time points increased or decreased respectively. For the uptake phase, it was assumed that the activity at the time of administration was zero and linearly increased to the first scan time. For the last phase, exponential decay with physical half-life was assumed from the last scan point to infinity to avoid any bias introduced by registration errors and redistribution of uptake at the voxel level. Voxel- *S* value kernels were generated with an application developed using the EGS++ class library within the general purpose EGSnrc Monte Carlo (MC) code [26, 27], previously validated by comparison with available resources [28]. Kernels for ^111^In and ^90^Y with 0.75 mm voxels in a soft-tissue homogeneous medium were generated. Simulations were carried out without variance reduction techniques and with 10^7^ histories to maintain the statistical uncertainty below 1% at central and nearest neighbouring voxels. The decay spectra used for the simulations were obtained from the RADTABS software [29].

Due to insufficient contrast between the tumour and the surrounding muscle tissue on the CT images, tumours were outlined in the SPECT images using a thresholding method using the PMOD software (PMOD Technologies). The threshold was varied until the tumour edge in the SPECT scan matched the visible tumour edge in the CT image. The thresholded final SPECT images were used to calculate the tumour masses assuming a density of 1 g/cm^3^ and the tumour activity using the calibration curve obtained from the phantom experiment. The tumour uptake, determined as the percentage injected activity per gram of tissue (%IA/g), was then calculated from the activity and mass of the tumour at the final scan time point. The tumour absorbed doses delivered by ^111^In-labelled mAbs and those that would have been delivered by ^90^Y-labelled mAbs were extrapolated to demonstrate the feasibility of pre-therapy-image-based treatment planning for therapeutic studies. The assumptions of equivalent injected activities, biological half-lives and uptake distributions for ^90^Y-labelled mAbs as for the ^111^In-labelled mAbs were made.

### Biodistribution study

The mice were euthanized by cervical dislocation immediately following the final scan: 96 h for four mice and 197 h for the remaining two mice. The tumours were dissected and weighed, and the radioactivity was measured in a WIZARD2 automatic gamma counter (PerkinElmer, UK). Tumour uptake (%IA/g), activity and mass were calculated. Comparisons between the three radiotracers were beyond the scope of this study.

### Comparison between imaging and biodistribution

The activity, mass and %IA/g in the tumour calculated from the last SPECT scan were compared with those obtained from the biodistribution study, which is considered to be the gold standard measurement.

### Statistical considerations

Median and range were used to describe continuous variables. Pearson’s correlation coefficients and regression analysis were used to quantify and identify relations between variables. Two-sided *P* values below 0.05 were considered significant.

## Results

### Immunoconjugate preparation and radiolabelling

The radiolabelling of ICR12 with ^99m^Tc, ^124^I and ^131^I has previously been reported [30–32], and this study demonstrates that it can also be efficiently labelled with ^111^In.

### Imaging study

#### Image quantification

For the attenuation/scatter phantom experiment, the mean counts in the sphere were on average 3% lower in the presence of the water-filled cylinder than with the air-filled cylinder.

**Fig. 1:**
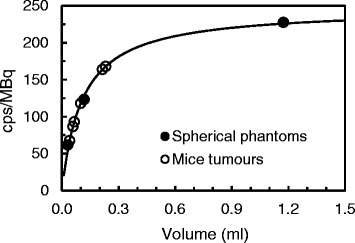
Calibration curve for a range of sphere sizes (*full circles*). The tumour volumes observed in the mice are also highlighted (*open circles*)

Figure 1 shows the calibration curves in units of counts per second (cps) per MBq. For the range of spherical phantoms and tumours studied, the calibration factor changed by a factor of 3.7 (62–227 cps/MBq) and 2.5 (67–168 cps/MBq), respectively.

#### Dosimetry

**Fig. 2:**
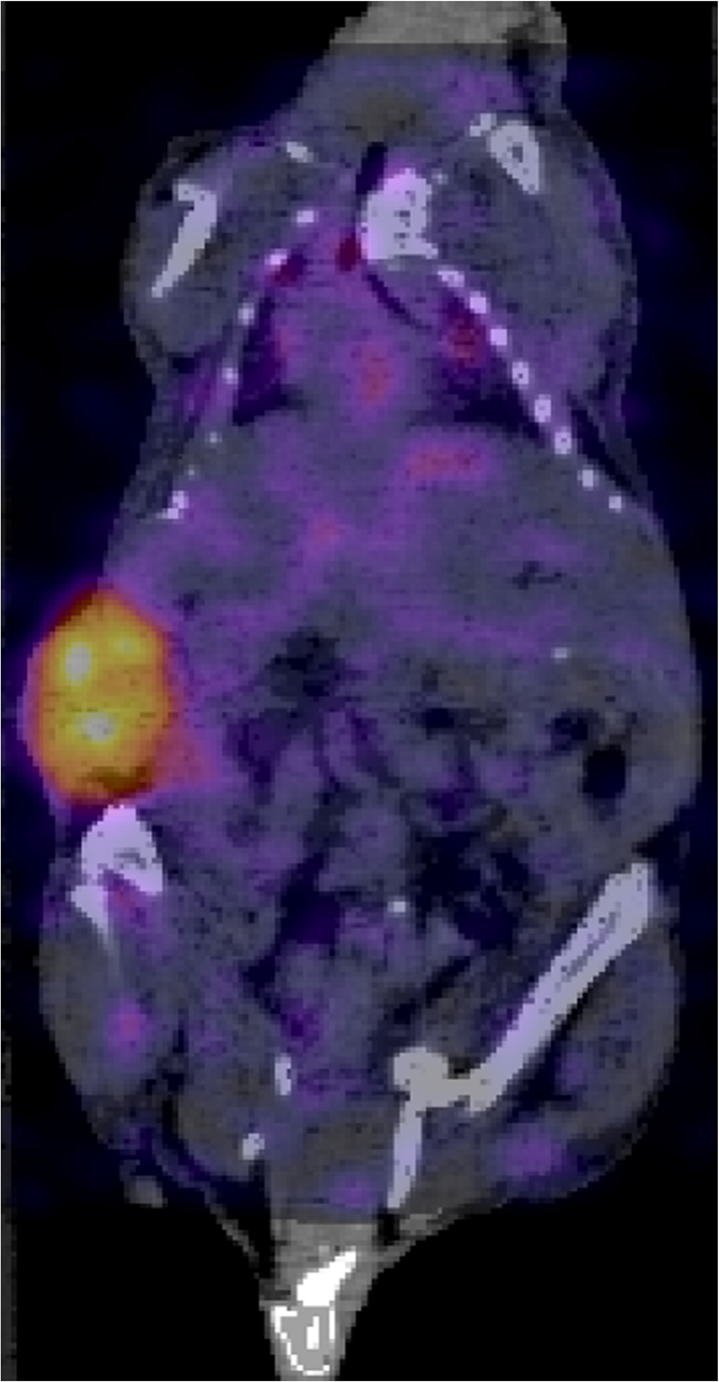
SPECT/CT slice through the tumour acquired at 96 h p.i. in mouse number 6 injected with ^111^In-DTPA-ICR12

**Fig. 3:**
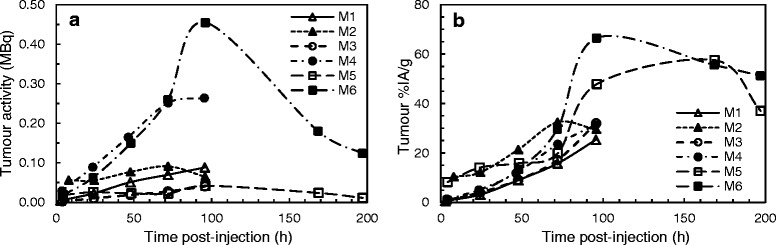
Time-activity (**a**) and %IA/g (**b**) curves obtained from sequential SPECT imaging of 6 mice. Mice were injected with ^111^In-DTPA-trastuzumab (M1), ^111^In-DTPA-ICR12 (M2, M3 and M6), ^111^In-DOTA-trastuzumab (M4, M5)

A representative SPECT/CT slice is shown in Fig. 2. A median tumour mass of 122 mg (range 28–264 mg) was obtained from the images, with a median activity of 0.077 MBq (range 0.011–0.264 MBq) and a median %IA/g of 32% (range 25–51%). The time-activity and time-uptake curves for the six mice are shown in Fig. 3. A median 36% (range 30–51%) of the activity was taken up by the tumour by 96 h pi. For mice 5 and 6, imaged at 197 h pi, 83 and 79% of the activity was taken up by the tumours, highlighting the importance of acquiring scans at later time points to improve the accuracy of the absorbed dose calculations. Mice 4 and 6 had the largest tumour sizes and showed a higher level of activity in the tumour as compared to the other mice (Fig. 3a). These differences were not observed in the %IA/g curves (Fig. 3b). The median tumour absorbed dose per injected activity (D/A) delivered by ^111^In-labelled antibodies was 52 cGy/MBq (range 36–69 cGy/MBq) and extrapolated for ^90^Y was 649 cGy/MBq (range 441–950 cGy/MBq) (Table 2).

**Table 2:** Mass, activity and uptake in the tumour determined from the biodistribution (bio) and imaging (im) studies and their relative percentage differences (diff), and delivered (^111^In) and extrapolated (^90^Y) tumour absorbed doses per injected activity (D/A) for the six mice included in the study

Mouse no.	Mass (mg)		Diff (%)	Activity (MBq)		Diff (%)	%IA/g		Diff (%)	D/A (cGy/MBq)	
	bio	im		bio	im		bio	im		^111^In	^90^Y
M1	100	152	52	0.108	0.088	–18	47	25	–46	36	441
M2	66	92	40	0.079	0.065	–18	51	29	–42	44	559
M3	59	61	3	0.041	0.041	–2	34	32	–6	52	572
M4	215	264	23	0.335	0.264	–21	49	31	–36	52	725
M5	41	28	–31	0.015	0.011	–21	33	37	14	65	745
M6	232	256	10	0.144	0.124	–14	66	51	–23	69	950

%IA/g was calculated at 96 h for M1–M4 and 197 h for M5 and M6

### Biodistribution study

A median tumour mass of 83 mg (range 41–232 mg) was obtained from the biodistribution data, with a median activity of 0.093 MBq (range 0.015–0.335 MBq) and a median %IA/g of 48% (range 33–66%).

### Comparison between imaging and biodistribution

**Fig. 4:**
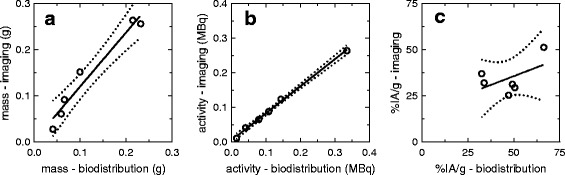
Tumour mass (**a**), activity (**b**) and %IA/g (**c**) calculated from the final SPECT images compared with those obtained from the biodistribution study. The 95% confidence interval in shown with *dotted* lines

Figure 4 shows the tumour mass, activity and %IA/g calculated from the final SPECT image for each mouse compared with the values determined by the biodistribution study. The median relative difference between the tumour mass determined from imaging and biodistribution was 17% (range –31 to 52%) (Fig. 4a). The activity in the tumour at the final scan time point was underestimated in all mice, with a median relative difference of –18% (range –21 to –2%) (Fig. 4b). A linear relationship was observed between imaging and biodistribution calculations of activity (*r* = 0.999, *P* < 0.0001) and mass (*r* = 0.977, *P* = 0.0008). When activity and mass were compounded together into the uptake calculation, no relationship between imaging and biodistribution uptake values was observed (*r* = 0.521, *P* = 0.29), with a median difference of –30% (range –46 to 14%) (Fig. 4c).

**Fig. 5:**
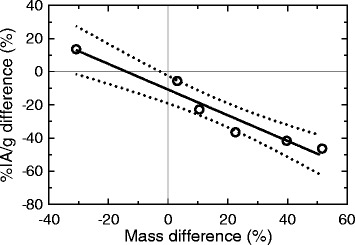
Relationship between the mass and %IA/g relative differences between imaging and biodistribution. The 95% confidence interval in shown with *dotted* lines

The relationship between the %IA/g and mass differences between imaging and biodistribution is shown in Fig. 5. In the ideal situation of perfect image quantification, if the same mass is determined from the imaging and the biodistribution data, no differences in %IA/g would be expected since the activity in the image would be accurately obtained. However, the %IA/g calculated from the SPECT image was still underestimated by 11% in comparison with the biodistribution calculation, obtained from the *y*-intercept of the linear regression line.

## Discussion

This study investigated the challenges associated with pre-clinical imaging and mouse-specific dosimetry and compared the mass, activity and %IA/g in the tumour obtained from the imaging and the ex vivo biodistribution data. A statistically significant correlation was observed between the tumour activity and mass as calculated from the biodistribution and imaging data at 96 and 197 h following injection of ^111^In-labelled mAbs targeting HER2-positive tumours. However, a correlation was not found between uptake as calculated from the imaging and the biodistribution data. Finucane et al assessed the quantification accuracy of ^111^In pre-clinical imaging and concluded that imaging could replace some dissection studies for the assessment of radiotracer biodistribution in mouse models [33]. Their conclusions were based on a comparison of the tumour activity calculated from the SPECT scan and the percentage of injected activity (%IA) determined from the biodistribution data and therefore did not include the influence of the mass into the %IA/g calculation. The discrepancies in the uptake calculations observed in our study are not fully understood, and more studies are needed to assess the reproducibility of measurements and to elucidate the interplay of the different variables in the calculation of uptake prior to the complete replacement of ex vivo biodistribution studies.

Accurate calculation of subcutaneous xenograft tumour volume is a key element of pre-clinical studies, as it is used as a metric to assess tumour growth and to quantify response to therapy, as well as for image quantification and dosimetry. Our results showed that for the range of tumours observed in this mouse cohort, the calibration factor to convert from counts to activity changed by a factor of 2.5, which can potentially lead to significant errors in the quantification of tumour activity, uptake and absorbed dose. These differences are likely due to partial volume effects, as the median tumour diameter observed in this study was 5.4 mm (range 4.3–7.6 mm), and the measured average axial spatial resolution for ^111^In was 2.1 (±0.4) mm. The investigation of the combined effects of attenuation and scatter showed only a 3% error on image quantification, which is small compared with the limitations of the spatial resolution and partial volume effects. Attenuation and scatter will play an important role for larger size rodents and low energy emitting radionuclides. For example, Hwang et al found that the measured concentration of activity in a volume of interest in the centre of a rat-sized water-filled cylinder was reduced by up to 50% for ^125^I and up to 25% for ^99m^Tc [9]. Our study was limited in that it only assessed the impact of attenuation/scatter, partial volume and limited spatial resolution. However, image quantification will also be affected by other factors. Optimisation of the number of iterations used for image reconstruction could lead to improved spatial resolution and thus reduce partial volume effects. Motion and image co-registration can also result in significant image blurring. It is therefore essential to standardise and harmonise the imaging protocols in pre-clinical imaging to decrease the variability in the calculation of tumour activity, %IA and absorbed dose, which are key parameters in the evaluation of novel radiotracers.

Tumour and organ masses can also have a significant impact on tabulated *S* values for organ and tumour-absorbed dose calculations. Several authors have studied the impact of various voxel-based mouse dosimetric reference models and found large variations in *S*-values due to the variability of the anatomical features [34–37]. Our study was based on convolution dosimetry, which accounts for heterogeneity in the uptake distribution at the voxel level. Tumour absorbed doses were calculated from outlining the absorbed dose distributions, neglecting tumour size changes that could occur following the administration of the radiotracer. Future studies could include magnetic resonance and/or ultrasound imaging to improve the accuracy and precision of tumour volume assessment [38, 39], which in turn will improve mass, activity, uptake and absorbed dose calculations.

The dosimetry methodology used in this study showed the feasibility of treatment planning in pre-clinical studies, where absorbed doses can be extrapolated for any radionuclide from a given uptake distribution. This could ease experimental planning for therapeutic radiotracers by informing the level of injected activity required to deliver a given absorbed dose, in particular for theragnostic agents, with the potential to reduce the number of animals used.

## Conclusions

This study demonstrates the need for multidisciplinary efforts to standardise imaging and dosimetry protocols in pre-clinical imaging before replacing ex vivo biodistribution studies. Accurate image quantification can have a large impact on improving the calculation of mass, activity, uptake and absorbed doses and therefore has the potential to improve treatment response studies and the comparison of novel radiotracers.
